# Supplementary figures and images for: Arginase-1+ Exosomes from Reprogrammed Macrophages Promote Glioblastoma Progression
Source: Int J Mol Sci. 2020 Jun 2;21(11):3990. doi: 10.3390/ijms21113990 (PMC7312363; doi:10.3390/ijms21113990)

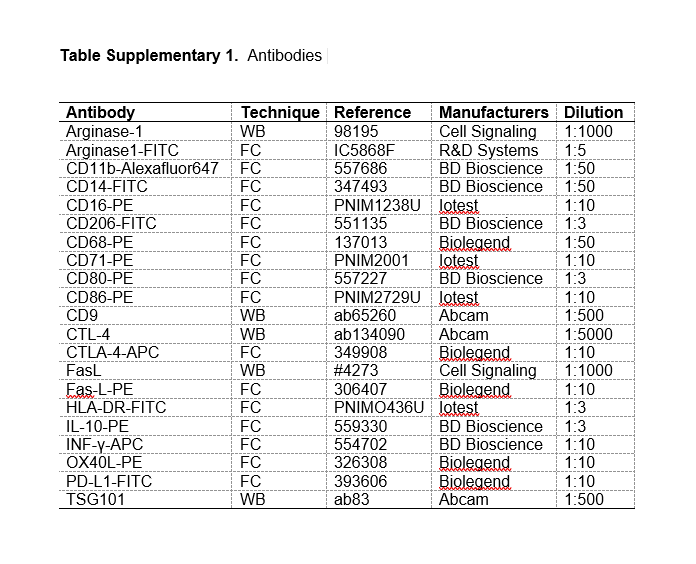

Supplement: Supplementary file 1 [file ijms-21-03990-s001.zip › ijms-795567-supp-final/Azambuja Table S2.tiff]

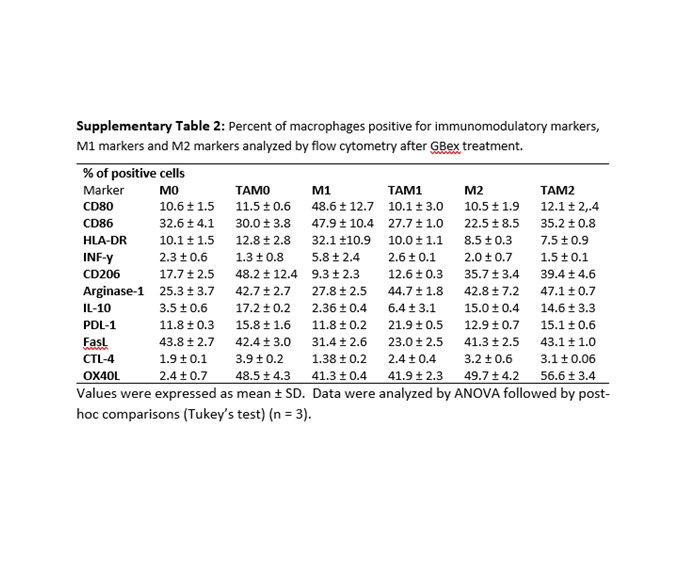

Supplement: Supplementary file 1 [file ijms-21-03990-s001.zip › ijms-795567-supp-final/Azambuja Table S1.tiff]

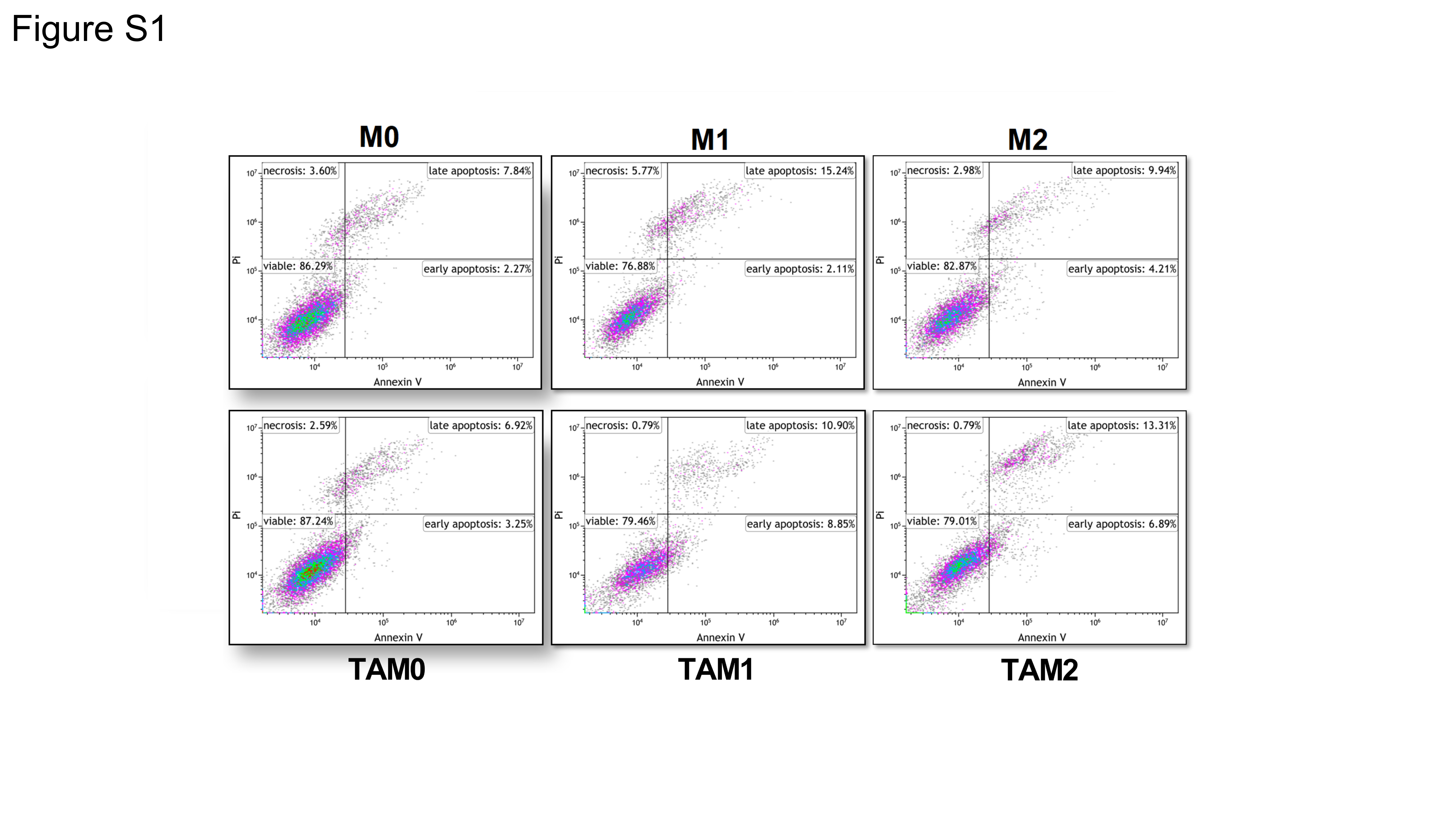

Supplement: Supplementary file 1 [file ijms-21-03990-s001.zip › ijms-795567-supp-final/Fig_S1.TIF]

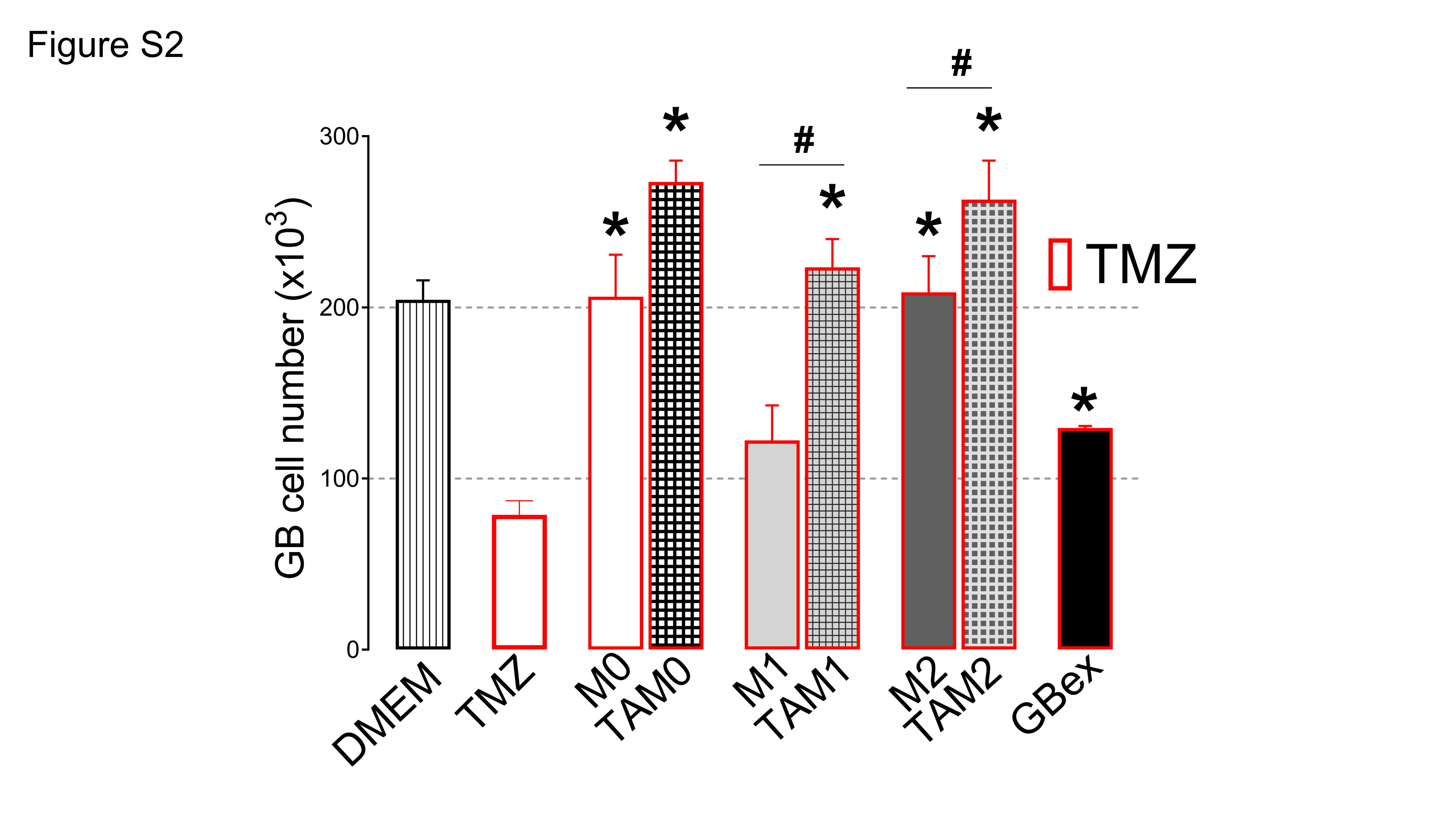

Supplement: Supplementary file 1 [file ijms-21-03990-s001.zip › ijms-795567-supp-final/Fig_S2.TIF]

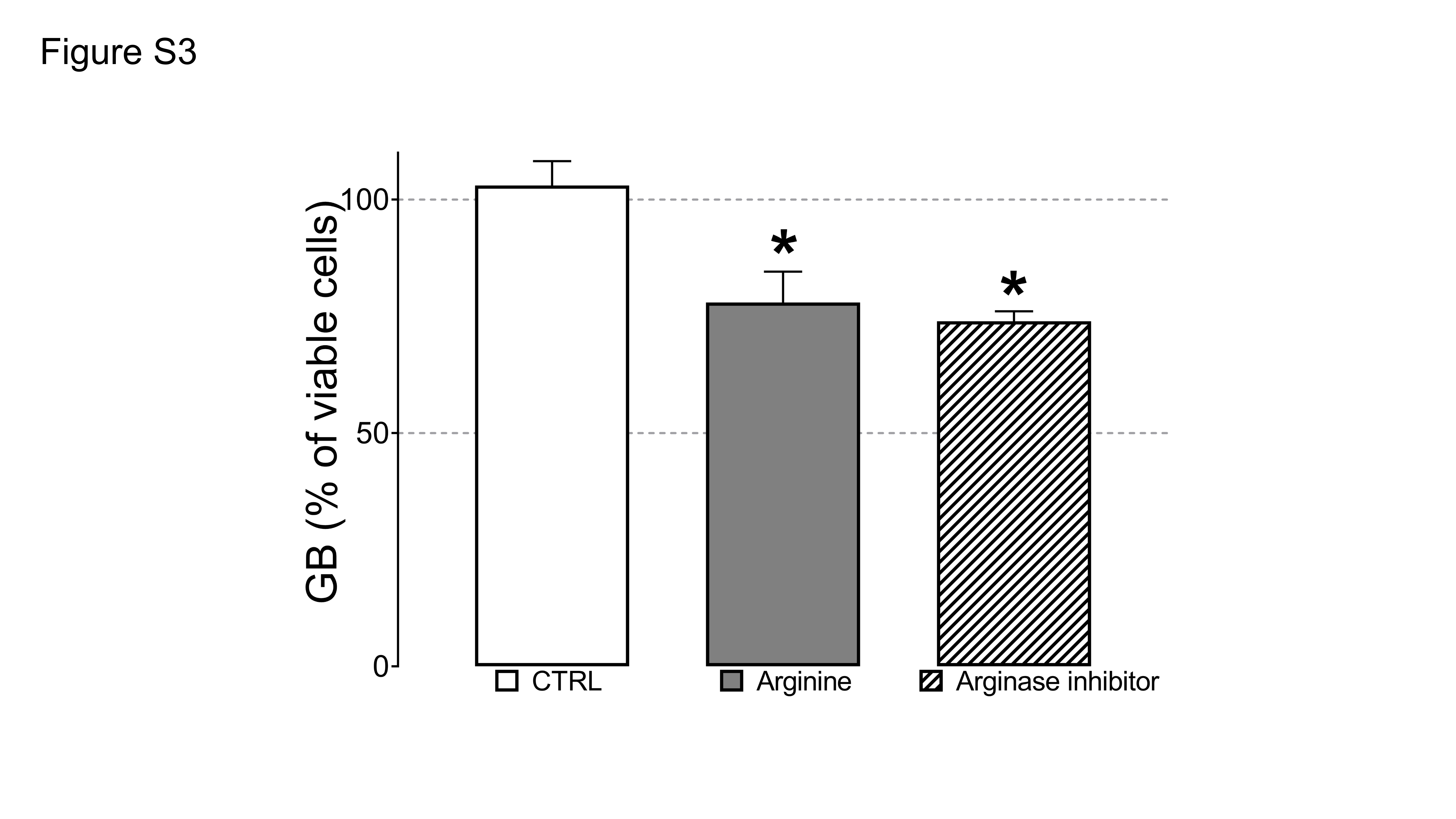

Supplement: Supplementary file 1 [file ijms-21-03990-s001.zip › ijms-795567-supp-final/Fig_S3.TIF]
